# Safety and Efficacy of Recombinant Fusion Protein Linking Coagulation Factor IX with Albumin (rIX-FP) in Previously Untreated Patients with Hemophilia B

**DOI:** 10.1055/s-0044-1781466

**Published:** 2024-03-26

**Authors:** Richard Lemons, Michael Wang, Julie Curtin, Lynda Mae Lepatan, Christoph Male, Flora Peyvandi, Mario von Depka Prondzinski, Rongrong Wang, William McKeand, Wilfried Seifert, Johannes Oldenburg

**Affiliations:** 1 Department of Pediatrics and Primary Children's Hospital, University of Utah, Salt Lake City, Utah, United States; 2 Hemophilia and Thrombosis Center, University of Colorado School of Medicine, Colorado, United States; 3 The Children's Hospital at Westmead, New South Wales, Australia; 4 Department of Health Research and Pediatrics, Cebu Normal University—Vicente Sotto Memorial Medical Center College of Medicine, Cebu, Philippines; 5 Department of Paediatrics, Medical University of Vienna, Vienna, Austria; 6 Fondazione IRCCS Ca' Granda Ospedale Maggiore Policlinico, Angelo Bianchi Bonomi Hemophilia and Thrombosis Center, Milan, Italy; 7 Department of Pathophysiology and Transplantation, Università degli Studi di Milano, Milan, Italy; 8 Werlhof Institute, Hannover, Germany; 9 CSL Behring, King of Prussia, Pennsylvania, United States; 10 CSL Behring, Marburg, Germany; 11 Institute of Experimental Hematology and Transfusion Medicine, University Hospital Bonn, Medical Faculty, University of Bonn, Bonn, Germany

**Keywords:** factor IX, hemophilia B, pediatric, previously untreated patients, prophylaxis

## Abstract

**Introduction** Recombinant fusion protein linking coagulation factor IX (FIX) with albumin (rIX-FP) has been shown to be an effective, well-tolerated treatment for patients with severe hemophilia B who had previously received factor replacement therapy. This study investigated the safety and efficacy of rIX-FP in previously untreated patients (PUPs).

**Methods** Patients with moderately severe/severe hemophilia B (≤2% FIX) previously untreated with FIX replacement products received rIX-FP (25–75 IU/kg) prophylaxis weekly or on-demand treatment over ≥50 exposure days (EDs). Primary outcomes were the number of patients who developed FIX inhibitors and mean incremental recovery (IR) following a 50 IU/kg dose of rIX-FP. Secondary outcomes included incidence of adverse events (AEs) and annualized bleeding rates (ABRs).

**Results** In total, 12 PUPs with a median age of 0 years (range, 0–11 years) were treated with rIX-FP for a median of 50 EDs (6/12 prophylaxis; 6/12 on-demand then prophylaxis). Overall, 11/12 patients did not develop FIX inhibitors; one 11-year-old patient developed an inhibitor against FIX after 8 EDs and was ultimately withdrawn. Mean (standard deviation) IR was 1.2 (0.4, *n* = 8) (IU/dL)/(IU/kg). Of the 137 treatment-emergent AEs recorded, five were attributed to rIX-FP. On the prophylaxis regimen, median ABR was 1.0 (range, 0–3.9, *n* = 12). No thromboembolic events or deaths occurred during the study.

**Conclusion** This study provides data to support the safety and efficacy of rIX-FP in PUPs requiring on-demand or prophylactic treatment for moderately severe/severe hemophilia B, consistent with results in previously treated patients. Overall, 1/12 patients developed an inhibitor against FIX.

## Introduction

Hemophilia B is an X-linked recessive inherited bleeding disorder resulting from a deficiency of coagulation factor IX (FIX). 1 Patients with hemophilia B are treated with FIX replacement therapies to raise FIX levels and to prevent bleeds in order to improve quality of life and allow patients to take part in their usual activities. 1 The current standard of care is regular prophylactic treatment with FIX replacement products. 1

Patients with hemophilia can be categorized according to their treatment history. Previously treated patients (PTPs) with hemophilia B are defined as those who have received FIX replacement therapy prior to another treatment regimen. Previously untreated patients (PUPs) with hemophilia B are those who have not received FIX replacement therapy, often young pediatric patients. This population is particularly vulnerable to severe bleeding episodes as patients become more active; therefore, it is essential that they receive effective preventative treatment from an early age. 1 Following one population pharmacokinetic (PK) model suggesting that a greater amount of time spent with FIX levels under 1% increases bleeding tendencies, recent guidelines now recommend a minimum FIX trough level of 3 to 5% for patients with severe hemophilia. 2 A secondary goal in pediatric treatment is to protect joints at an early age from damage caused by spontaneous bleeding episodes. 1 Improving FIX trough levels through prophylactic factor replacement can also prevent joint hemarthrosis and allow an active lifestyle. 3 One of the most prominent challenges in hemophilia treatment is the development of inhibitors. PUPs are at greater risk of developing inhibitors to FIX, which can also trigger allergic and anaphylactic reactions and affect treatment outcomes negatively. 1 4 5

Recombinant fusion protein linking recombinant coagulation FIX with recombinant albumin (rIX-FP; IDELVION; CSL Behring, Marburg, Germany) is an extended half-life FIX replacement product indicated for use in patients with hemophilia B. 6 In previous studies, rIX-FP has shown a favorable safety profile and effective treatment of bleeds in adult and pediatric patients who had been previously treated with FIX replacement therapy. 7 8 9 rIX-FP has also demonstrated improved PK profiles in comparison with standard half-life factor replacement products, which offers improved protection against bleeds. 10 11 This also allows the option to extend dosing intervals up to every 21 days in well-controlled adult patients, to reduce the burden on patients and their families without compromising efficacy. 9

The aim of this study was to evaluate the safety and efficacy of rIX-FP in PUPs with hemophilia B. The primary outcomes were to evaluate the immunogenicity of rIX-FP as measured by new cases of inhibitors against FIX and the mean incremental recovery (IR) of a 50 IU/kg dose of rIX-FP. Secondary outcomes included further evaluation of safety through the incidence of adverse events (AEs) and AEs related to rIX-FP over the study period. The efficacy of rIX-FP was also evaluated as a secondary objective, via total annualized bleeding rate (ABR), annualized spontaneous bleeding rate (AsBR), and rIX-FP consumption during routine prophylaxis. Additional exploratory outcomes included investigator assessment of efficacy during major bleeding episodes, number of rIX-FP infusions required, further evaluation of rIX-FP efficacy for prophylaxis in PUPs, and evaluation of PK profiles following a single 50 IU/kg dose of rIX-FP.

## Methods

### Study Conduct

The study was approved by an institutional review board/independent ethics committee at each participating center, registered at www.clinicaltrials.gov (NCT02053792), and performed in accordance with Good Clinical Practice, the Declaration of Helsinki, and local regulatory requirements. Written informed consent was obtained before undergoing any study-specific procedures. Consent could be withdrawn at any time.

### Study Participants

Male patients aged ≤18 years, with a diagnosis of moderately severe/severe hemophilia B (FIX activity ≤2%, according to European Medicines Agency guidelines, 2011 12 ) were eligible for enrollment in this study. Patients had no previous exposure to FIX replacement products, although exposure to other blood products was permitted. Patients also had a confirmed history of no FIX inhibitor formation and were willing to adhere to study protocols.

### Study Design

This study reports the findings of a phase 3b, prospective, multicenter study of PUPs with hemophilia B. At enrollment, patients were encouraged to begin rIX-FP prophylaxis once weekly (25 to 50 IU/kg initially, up to 75 IU/kg), but this was not a requirement and on-demand treatment was also permitted. On-demand treatment with rIX-FP was provided for bleeding episodes with a target FIX activity of 40 to 80% at a dosage of 35 to 75 IU/kg, determined by the investigator based on the patient's PK data. At the end of the first 12-month trial period, patients were then required to be on a weekly prophylaxis regimen with rIX-FP until the completion of 50 exposure days (EDs). Following 50 EDs, patients were allowed to continue with rIX-FP prophylaxis at weekly intervals until the end of the study. A PK evaluation of mean IR with 50 IU/kg rIX-FP was performed on the day of the first dose, if feasible, or during the study after at least 7 days washout when the patient was in a nonbleeding state.

If any patient required minor nonemergency surgery during the study period, they could enroll into the optional surgery substudy. Efficacy and safety data during and after surgery were recorded, including rIX-FP dosage, total consumption, investigator's assessments of blood loss, overall efficacy, transfusion requirements, and AEs.

### Study Objectives

#### Safety Endpoints

The primary endpoint for this study was the development of inhibitors against FIX. Secondary endpoints included occurrence of AEs and AEs related to rIX-FP. The number and percentage of patients who had ≥1 treatment-emergent adverse event (TEAE) and/or serious adverse event (SAE) were also recorded. Exploratory endpoints were the incidence of patients who developed antibodies against rIX-FP or Chinese hamster ovary (CHO) host cell proteins, total EDs, and number of patients who developed inhibitors against FIX during the surgery substudy.

#### Efficacy Endpoints

All efficacy analyses were either secondary or exploratory. Efficacy assessments for routine prophylaxis included total and traumatic ABRs, AsBRs, annualized joint bleeding rates, total consumption of rIX-FP, and unknown bleeding episodes. All bleeding episodes were evaluated and diagnosed by the treating physician, based upon their own professional experience, and were reported as spontaneous, traumatic, or unknown. For treated bleeds, treatment was considered successful by the physician if hemostasis was achieved with one or two infusions.

#### Pharmacokinetic Endpoints

PK assessments used a noncompartmental analysis conducted on plasma FIX. The PK endpoints observed were mean IR, maximum concentration ( *C*_max_ ) and steady-state FIX activity. Samples for IR were collected 30 minutes after dosing.

### Statistical Analysis

Data were summarized using descriptive statistics unless otherwise stated. In general, data were summarized by study period and age group, and included the total number of subjects in the study population. Bleeding episodes during the PK period were included in the analysis if the bleed was treated with rIX-FP. Bleeding episodes during the surgical period were excluded from analysis. Two patients had PK evaluations performed within 2 days of a bleeding episode and were, therefore, excluded from the PK population.

## Results

### Study Population

In total, 15 patients with FIX activity ≤2% were screened, 14 patients were enrolled, and 12 patients were treated in this study. Four of the fourteen patients enrolled ultimately discontinued from the study: one due to physician's decision before receiving study treatment and three who discontinued during treatment (two patients withdrew themselves from the study, one patient had to discontinue due to hypersensitivity reactions during on-demand treatment with 100 IU/kg rIX-FP following development of a high-titer FIX inhibitor). Overall, eight patients accumulated a minimum of 50 EDs as per the protocol.

In total, 12 patients with a median age of 0 years (range 0–11 years) were treated in this study; the majority of patients were <6 years old ( *n* = 11) and one patient was aged 11 years old ( Table 1 ). The majority of patients were from Europe (41.7%). No patients had a family history of inhibitors against FIX. Data on genetic mutations were available in three patients; two patients <6 years old had exon 8 mutations (C.1235g > a, P.Gly412glu) and Nm_000133.3( *F9* ):c(1025c > t); (1025 = ), and an 11-year-old patient who later developed an inhibitor had a large deletion of exons 7 and 8 of the *F9* gene.

**Table 1:** Patient characteristics

Characteristics	<6 years ( *n* = 11)	≥6–12 years ( *n* = 1)	Total ( *n* = 12)
Mean time since diagnosis (SD), months	5.2 (4.3)	21.5	6.6 (6.2)
Median age (min, max), years	0.0 (0, 1)	11.0	0.0 (0, 11)
Mean weight (SD), kg	8.9 (2.3)	28.0	10.5 (5.9)
Race, *n* (%)			
White	10 (90.9)	0	10 (83.3)
Asian	0	1 (100.0)	1 (8.3)
Other	1 (9.1)	0	1 (8.3)
Geographic region, *n* (%)			
North America	4 (36.4)	0	4 (33.3)
Europe	5 (45.5)	0	5 (41.7)
Oceania a	2 (18.2)	1 (100.0)	3 (25.0)
Time since first diagnosis mean (SD), months	5.2 (4.3)	21.5	6.6 (6.2)

Abbreviation: SD, standard deviation.

a Oceania includes Australia and Philippines

### Dosing Regimens

**Fig. 1:**
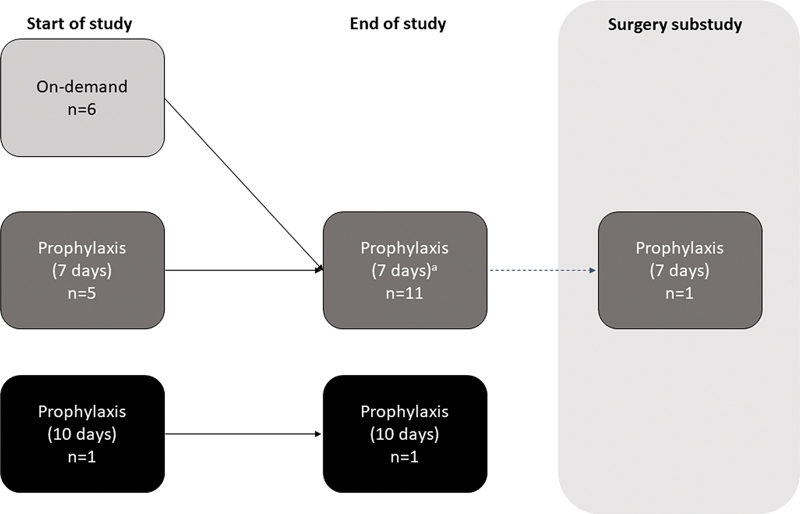
Study flow diagram. *n* = number of patients. ^a^ All patients ended the study on the prophylactic regimen, but the 11-year-old patient who developed an inhibitor in the 7-day cohort was switched to an off-protocol intensified regimen (dose increased from 50 to 100 IU/kg).

At the time of study initiation, 6/12 patients were initiated on rIX-FP prophylaxis from enrollment, with 5/6 patients being initiated on a 7-day regimen and one patient on a 10-day regimen ( Fig. 1 ). The remaining 6/12 patients received on-demand treatment with rIX-FP for 1.9 to 12.3 months prior to switching to a 7-day prophylaxis regimen ( Fig. 1 ). Patients had a median of 50 EDs over 680 days (mean of 68.3 EDs) including the PK and surgery periods (median of 47 EDs during prophylaxis regimen), with a mean total rIX-FP dose of 3,298 IU/kg ( Table 2 ). During the study, 11/12 patients were being treated with rIX-FP on a 7-day regimen and one patient was on a 10-day regimen ( Fig. 1 ).

**Table 2:** Treatment regimen time and dose

	<6 years ( *n* = 11)	≥6–12 years ( *n* = 1)	Total ( *n* = 12)
Median time on study (min, max), days	680.0 (143, 1,002)	783.0	680.0 (143, 1,002)
Median exposure days (min, max)	50.0 (22, 146)	39.0	50.0 (22, 146)
Mean total dose during study (SD), IU/kg	3,321.3 (1,859.8)	3,043.7	3,298.1 (1,775.1)
Mean time on regimen (SD), days			
On-demand period ( *n* = 6)	271.2 (127.1)	0	271.2 (107.1)
Prophylaxis total ( *n* = 12)	497.5 (289.6)	94.0	463.8 (299.7)
7-day regimen ( *n* = 11)	510.3 (301.9)	94.0	472.5 (312.7)
10-day regimen ( *n* = 1)	369.0	0	369.0
Intensified treatment ( *n* = 1)	0	616.0	616.0

Abbreviation: SD, standard deviation.

### Safety

During the study, 11/12 patients did not develop FIX inhibitors over a 24-month study period. The 11-year-old patient developed an initially low-titer (0.58 BU/mL) FIX inhibitor after 8 EDs of rIX-FP and experienced a mild hypersensitivity reaction related to rIX-FP. One month later, the patient received the last dose of routine 50 IU/kg rIX-FP prophylaxis, and 14 days postinfusion the inhibitor levels had increased to a high-titer of 5.6 BU/mL (6 EDs since the low-titer inhibitor was recorded). In response to the inhibitor development, the patient received an off-protocol intensified treatment regimen of 100 IU/kg every 7 days (as agreed with the Independent Data Monitoring Committee) for approximately 2 months but this resulted in a further increase of the inhibitor titer. The patient discontinued the intensified regimen after 8 exposures due to difficulties complying with the intense schedule. He was switched to on-demand treatment for a period of approximately 1 year, with bleeding episodes successfully treated with doses of 100 IU/kg rIX-FP. The patient was ultimately withdrawn from the study by the investigator due to two mild hypersensitivity reactions during rIX-FP infusions to treat a joint bleed. During the first reaction, symptoms included pallor, fever, respiratory rate increase, and abdominal pain, which were interpreted by the investigator as an allergic reaction related to rIX-FP. The patient then experienced a nonserious, mild allergic reaction following the next rIX-FP infusion, after which he was withdrawn. The patient was identified as having *F9* genetic deletions as reported above and had previously received transfusions with packed red blood cells but had no further medical history related to his hemophilia. No patients developed nonneutralizing antidrug antibodies against rIX-FP or CHO cell-derived proteins.

Overall, excluding AEs occurring during the surgery period, 11/12 patients experienced 135 TEAEs; most TEAEs were mild and most resolved. The most common TEAE was of infection or infestation (57/135). The majority of TEAEs (109/135) occurred in patients receiving the prophylaxis regimen ( Table 3 ). Two patients had five TEAEs related to rIX-FP, of which two events occurred during prophylaxis and the remaining three during off-protocol intensified treatment. One TEAE related to rIX-FP of a rash was recorded in a patient <6 years old after four doses on the 7-day prophylaxis regimen. The remaining four TEAEs related to rIX-FP occurred in the 11-year-old patient who developed an inhibitor; these included three mild TEAEs of hypersensitivity (2/3 occurred during intensified treatment) and one SAE of anti-FIX antibody increase. Five patients had five SAEs, which were recorded as follows: influenza, device-related infection, head injury, pneumonia, and anti-FIX antibody increase. Three patients had nine AEs of special interest, which were recorded as hypersensitivity (including rash and urticaria). There were no local reactions associated with rIX-FP treatment, and no safety concerns were indicated in laboratory parameters or vital signs. No deaths, cases of anaphylaxis, or cases of thrombotic events occurred during the study period.

**Table 3:** Overview of treatment-emergent adverse events occurring during prophylaxis

Category	Prophylaxis regimen, *n* = 12	
	PUPs, n (%)	Events
Any TEAEs	11 (91.7)	109
Intensity		
Mild	11 (91.7)	86
Moderate	5 (41.7)	21
Severe	2 (16.7)	2
TEAEs related to rIX-FP	2 (16.7) a	2 a
Any SAEs	3 (25) b	3

Abbreviations: PUP, previously untreated patient; rIX-FP, recombinant fusion protein linking factor IX with albumin; SAE, serious adverse event; TEAE, treatment-emergent adverse event.

a The two related TEAEs were recorded as hypersensitivity in the 11-year-old patient who later developed an inhibitor and development of a rash on lower legs and forearms in a second patient. The rash was considered mild in intensity, resolved within 4 days, and prophylaxis continued in this patient.

b Including the patient who developed an inhibitor (patient had a large gene deletion) and discontinued the study.

### Efficacy

The mean (standard deviation [SD]) dose of rIX-FP for routine prophylaxis treatment was 45.9 (4.5) IU/kg. The median monthly dose of rIX-FP for patients who had received prophylaxis treatment for >6 months was 192.5 IU/kg ( Table 4 ). Overall, 8/12 patients had a prophylaxis compliance of ≥80%. Consumption rates per bleeding episode for on-demand and for prophylaxis regimens are available in Supplementary Table S1 (available in the online version).

**Table 4:** Consumption of rIX-FP as prophylaxis treatment for subjects on prophylaxis treatment for >6 months (>183 d)

	7-day regimen ( *n* = 9)	10-day regimen ( *n* = 1)	Total ( *n* = 10)
Monthly dose (IU/kg)			
* n*	9	1	10
Median	195.9	132.1	192.5
Min, Max	171.8, 215.6	132.1, 132.1	132.1, 215.6
Yearly dose (IU/kg)			
* n*	9	1	10
Median	2,350.7	1,584.8	2,309.7
Min, Max	2,062.1, 2,587.1	1,584.8, 1,584.8	1,584.8, 2,587.1

Note: Consumption of rIX-FP in this table includes all doses administrated during prophylaxis periods.

On the prophylaxis regimen, 12 patients had a median ABR of 1.0 (range 0–3.9), with 9/12 patients recording an AsBR of 0. The ABR of 3.9 occurred in the patient who developed an inhibitor, who had 94 days on prophylaxis prior to developing FIX inhibitors ( Supplementary Table S2 , available in the online version). Notably, one patient who had >6 months of prophylaxis treatment on a 10-day regimen had a recorded ABR of 1.0 ( Supplementary Table S3 , available in the online version). The ABRs based on treated or total bleeding episodes for individual subjects on prophylaxis treatment for >6 months are available in Table 5 . In the 10 patients who were treated with prophylaxis for >6 months, median ABR was 1.1 ( Table 5 ), and median total yearly consumption was 2309.7 IU/kg.

**Table 5:** Summary of total annualized bleeding rates for patients on prophylactic treatment for >6 months (>183 days)

	ABRs based on treated bleeding episodes	ABRs based on all bleeding episodes
7-day regimen ( *n* = 9)		
* n*	9	9
Mean (SD)	0.6 (0.7)	1.2 (1.1)
Median	0.00	1.2
Min, Max	0.0, 1.5	0.0, 3.1
10-day regimen ( *n* = 1)		
* n*	1	1
Mean (SD)	1.0 (NC)	1.0 (NC)
Median	1.0	1.0
Min, Max	1.0, 1.0	1.0, 1.0
Total ( *n* = 10)		
* n*	10	10
Mean (SD)	0.6 (0.7)	1.2 (1.0)
Median	0.5	1.1
Min, Max	0.0, 1.5	0.0, 3.1

Abbreviations: ABR, annualized bleeding rate; NC, not calculable; SD, standard deviation.

**Table 6:** Number of rIX-FP infusions required to achieve hemostasis following spontaneous bleeding events

	On-demand ( *n* = 6)	7-day prophylaxis ( *n* = 11)	10-day prophylaxis ( *n* = 1)	Prophylaxis total ( *n* = 12)	Intensified treatment ( *n* = 1)	Total ( *n* = 12)
Number of PUPs with ≥1 bleeding episodes, *n* (%)	1 (16.7)	3 (27.3)	0	3 (25.0)	1 (100.0)	4 (33.3)
Number of bleeding episodes requiring treatment	1	4	0	4	11	16
Number of injections to treat a bleeding episode, *n* (%)						
1 injection	1 (100.0)	2 (50.0)	0	2 (50.0)	10 (90.9)	13 (81.3)
1–2 injections	1 (100.0)	4 (100.0)	0	4 (100.0)	10 (90.9)	15 (93.8)
2 injections	0	2 (50.0)	0	2 (50.0)	0	2 (12.5)
>2 injections	0	0	0	0	0	0
Unknown	0	0	0	0	1 (9.1)	1 (6.3)

Abbreviation: PUP, previously untreated patient.

**Table 7:** PK parameters and FIX activity

Parameter	<6 years	≥6 to ≤12 years	Total
Baseline-corrected PK parameters, mean (SD) after a single rIX-FP dose (50 IU/kg)			
PK population	*n* = 7	*n* = 1	*n* = 8
*C*_max_ (IU/dL)	63.17 (15.8)	39.90	60.26 (16.8)
IR (IU/dL)/(IU/kg)	1.29 (0.4)	0.80	1.23 (0.4)
Steady-state FIX activity on 7-day prophylaxis regimen			
Safety population a	*n* = 7	–	*n* = 7
Mean steady-state FIX activity (SD), IU/dL	12.49 (6.1)	–	12.49 (6.1)
Median steady-state FIX activity (range), IU/dL	11.85 (2.7–31.3)	–	11.85 (2.7–31.3)

Abbreviations: C _max_ , maximum concentration; IR, incremental recovery; *n* , number of PUPs receiving 50 IU/kg; PK, pharmacokinetic; PUP, previously untreated patient; rIX-FP, recombinant fusion protein linking coagulation factor IX with albumin.

a 7/12 patients from the safety population had steady-state values derived per protocol definition.

No major bleeds were reported in any patient during the study; therefore, the investigator assessment of rIX-FP efficacy during major bleeding episodes did not take place. Excluding PK and surgery study periods, 24 joint bleeds occurred in 6/12 patients (14 were spontaneous, seven were traumatic and three were unknown); 23/24 joint bleeds required treatment and 9/24 joint bleeds occurred in four patients treated on the 7-day prophylaxis regimen. The patient who developed an FIX inhibitor experienced 13 joint bleeds during intensified treatment. Across study periods, excluding the PK and surgery periods, 44 bleeds requiring treatment occurred in 12 patients, of which 20 bleeding events took place in patients on the 7-day prophylaxis regimen (5/20 occurred ≤3 days and 15/20 occurred >3 days and ≤7 days after most recent prophylactic treatment). Overall, excluding PK and surgery periods, 37 bleeds were treated with rIX-FP. Spontaneous bleeds were successfully controlled with one or two infusions of rIX-FP in 93.8% of occurrences ( Table 6 ).

### Pharmacokinetics

PK profiles were evaluated for 8/12 patients who had sufficient FIX activity measurements following a single rIX-FP infusion of 50 IU/kg ( Table 7 ). The mean (SD) IR was 1.2 (0.4) (IU/dL)/(IU/kg), and the mean (SD) *C*_max_ was 60.3 (16.8) IU/dL. For the patient who developed an inhibitor, IR was 0.8 (IU/dL)/(IU/kg) prior to inhibitor development.

For the overall safety population, the mean (SD) steady-state FIX activity was 12.5 (6.1) IU/dL for the seven patients with available data.

### Surgery Substudy

One patient underwent a surgical procedure for venous access port placement and took part in the surgical substudy. During the 8-day surgical period, the patient experienced a bleeding event which was treated with 50 IU/kg rIX-FP. The patient experienced one TEAE of mild soft tissue swelling, which was resolved. The total rIX-FP consumption was 252 IU/kg, which was administered in four doses during the 8-day surgical period. The predicted blood loss estimated by the investigator before surgery and the actual blood loss during surgical drainage was 5 mL. No transfusion of blood products was reported during the surgical period, and no further information was reported by the investigator.

Two further patients underwent three surgical procedures but were not enrolled into the surgical substudy. The first patient underwent two surgical procedures for the insertion of single vascular access port and insertion of bilateral ear tubes. No bleeding events were experienced during the 7-day surgical period. The patient was administered a total of 198 IU/kg rIX-FP in three doses for the port insertion and 147 IU/kg in two doses for the ear tube insertion. The second patient underwent a surgical procedure for sagittal suture craniosynostosis. No bleeding events or AEs were experienced during the 17-day surgical period. The patient was administered a total of 490 IU/kg rIX-FP for the revision of the sagittal suture craniosynostosis.

## Discussion

The data from this study in 12 PUPs further confirm the safety and efficacy of rIX-FP for routine prophylaxis factor replacement therapy or on-demand treatment of patients with hemophilia B. Overall treatment was well tolerated, with no deaths or local reactions attributed to rIX-FP.

The primary endpoint for this study was the number of PUPs who developed inhibitors following the onset of prophylaxis with rIX-FP. Herein, we report that only one out of 12 patients (8.3%) experienced FIX inhibitor formation, an incidence similar to that previously reported in PUPs with hemophilia B (9.3%). 13 The 11-year-old patient who developed an inhibitor was ultimately discontinued after 39 EDs due to hypersensitivity reactions to treatment. This patient had large deletions within exons 7 and 8 of the *F9* gene, which have previously been identified as a risk factor for inhibitor development, as noted in a recent study which found that patients with large structural deletions had a higher risk (33.3%) of inhibitor development. 13

The second primary endpoint for this study was the estimated mean (SD) IR (IU/dL)/(IU/kg) for patients receiving a 50 IU/kg dose of rIX-FP, which was comparable to previous studies in adult patients (mean IR 1.23 vs. 1.38) and is higher than that observed with alternative FIX products (mean IR: 0.94 rFIX, 1.10 pdFIX). 10 The PK profiles available in this study demonstrate that a mean steady-state FIX activity of >10% is achievable with rIX-FP treatment. Such trough levels are above the target currently set out in the World Federation of Hemophilia guidelines. 1 8 9 The results from this study are similar to those seen in previously treated pediatric patients treated with rIX-FP, when mean steady-state FIX activity levels reached 15.3 and 15.0% for patients aged <6 years and ≥6–12 years on 7-day regimens (25–50 IU/kg), respectively. 8 Another study of adult PTPs treated with rIX-FP reported mean steady-state FIX activity levels of 22.0% in patients on a 7-day regimen (35–50 IU/kg) and 19.8% in patients on a 10-day regimen (50–75 IU/kg). 9 Previously, the concept of extravascular distribution of FIX has been considered as a reason for the differing PK values seen with different FIX replacement products; however, the potential impact on clinical outcomes is currently hypothetical. 14

Effective bleed control is of particular significance in pediatric patients for the preservation of joint status during high activity at a young age. 1 Routine prophylaxis with rIX-FP provided effective bleed protection in PUPs, with 9/12 patients maintaining an AsBR of 0 and no reports of major bleeds. Spontaneous bleeding episodes were treated effectively with one to two doses of rIX-FP in 93.8% of cases, which is comparable to the high levels of bleeding control seen in previous studies of adult and pediatric PTPs. 7 15 While patients were on the prophylactic regimen, only four bleeding episodes requiring treatment occurred.

TEAEs were mostly mild or moderate, not treatment related, and were resolved during the study. The safety profile of rIX-FP use in PUPs was similar to that seen previously in studies of PTPs. 7 10 In the current study, 25% of patients receiving rIX-FP developed treatment-emergent SAEs during the prophylaxis period. In the 10 patients who completed >6 months of prophylactic treatment, median ABR was 1.1 and median yearly consumption was 2309.7 IU/kg. A similar study of 27 PUPs treated with an alternative recombinant FIX product (rFIXFc) showed that 69.7% patients developed treatment-emergent SAEs, with a median ABR of 1.2 and median yearly consumption of 3,175.0 IU/kg. 16 In the rFIXFc study, one patient with a nonsense mutation developed a low-titer inhibitor after 11 EDs and was discontinued after a hypersensitivity reaction; however, no high-titer inhibitors were detected. 16 This inhibitor development was the only treatment-emergent SAE considered related to rFIXFc treatment, similar to the results of the current study. 16

In a study of 37 PUPs and minimally treated patients who received the FIX replacement product N9-GP as preprophylaxis, prophylaxis, or on-demand therapy, 2/37 PUPs (5.4%) developed high-titer inhibitors after 4 EDs. 17 Median ABRs were low (0.0 across all regimens) with an estimated mean FIX trough activity of 15.0% and only 37.8% of patients developed SAEs. Median yearly N9-GP prophylaxis consumption was 2,280.8 IU/kg. 17

The main limitation of the present study is its small sample size, which remains to be generalized to the wider population. 16 17 Further studies on data collected from registries with large, diverse populations should be conducted to support the results.

## Conclusions

In conclusion, this open-label study provides data to support the safety and efficacy of rIX-FP in PUPs requiring on-demand or prophylactic treatment for moderately severe/severe hemophilia B. One patient out of 12 developed an inhibitor to FIX. The results generated by this study are consistent with previously reported results of rIX-FP use in PTPs.

## Notes

**What is Known on This Topic?**

- Prophylactic factor replacement is the standard of care treatment for patients with hemophilia.
- In previous studies, factor replacement with rIX-FP has shown favorable safety and efficacy profiles in previously treated patients with hemophilia B.
- Previously untreated patients with hemophilia are often very young and particularly vulnerable to severe bleeding episodes and the development of inhibitors following treatment with factor replacement.

**What Does This Paper Add?**

- This study evaluates the safety and efficacy of rIX-FP in PUPs with hemophilia B.
- Results reported include inhibitor formation, pharmacokinetic data, bleeding rates, and consumption data.
