# Supplementary material for: Safety and Efficacy of Recombinant Fusion Protein Linking Coagulation Factor IX with Albumin (rIX-FP) in Previously Untreated Patients with Hemophilia B
Source: TH Open. 2024 Mar 26;8(1):e155–63. doi: 10.1055/s-0044-1781466 (PMC10965291; doi:10.1055/s-0044-1781466)
Supplement: Supplementary file 1 — Supplementary Material [file 10-1055-s-0044-1781466-s24010003.pdf]

Supplementary Information

Supplementary Table S1 Consumption of rIX-FP for bleeding episodes

	On-demand period	7-day regimen period	10-day regimen period	Total
Number of patients with treated bleeding episodes				
	4	6	1	10
Number of treated bleeding episodes				
	6	14	1	21
Total dose [IU/kg] per bleeding episode				
n	6	14	1	21
Median	50.2	57.6	50.0	50.4
Min, Max	34.0, 102.0	35.2, 303.6	50.0, 50.0	34.0, 303.6
Dose [IU/kg] per spontaneous bleeding episode				
n	1	4	0	5
Median	50.4	77.9	N/A	64.2
Min, Max	50.4, 50.4	49.9, 100.4	N/A	49.9, 100.4
Dose [IU/kg] per traumatic bleeding episode				
n	5	9	1	15
Median	50.0	51.0	50.0	50.2
Min, Max	34.0, 102.0	47.4, 303.6	50.0, 50.0	34.0, 303.6
Dose [IU/kg] per unknown bleeding episode				
n	0	1	0	1
Median	N/A	35.2	N/A	35.2
Min, Max	N/A	35.2, 35.2	N/A	35.2, 35.2

n, number of bleeding episodes; N/A, not applicable.

Supplementary Table S2 Annualized bleeding rate of bleeding episodes requiring treatment, per patient

	On-demand period		7-day prophylaxis		10-day prophylaxis	
Patient ID	Spontaneous	Total	Spontaneous	Total	Spontaneous	Total
Patient 1	0	0	0	0	–	–
Patient 2	0	0	0	0	–	–
Patient 3	0	2.1	0	0	–	–
Patient 4	–	–	0	1.1	–	–
Patient 5	–	–	–	–	0	1.0
Patient 6	–	–	0	1.0	–	–
Patient 7	–	–	0.4	1.5	–	–
Patient 8[a]	–	–	3.9	3.9	–	–
Patient 9	–	–	0.8	1.5	–	–
Patient 10	–	–	0	2.7	–	–
Patient 11	1.2	3.7	0	0	–	–
Patient 12	0	1.0	0	0	–	–

[a]The 11-year-old patient who developed an FIX inhibitor. During intensified treatment after inhibitor development, the total annualized bleeding rate was 9.49 and the annualized spontaneous bleeding rate was 6.52.

 © 2024. The Author(s).

Supplementary Table S3 Total annualized bleeding rates for patients on prophylactic treatment for >6 months (>183 days), per patient

Patient ID	Period		Bleeding episodes		ABR	
	Regimen	Duration (days)[a]	Treated	Not treated	Treated	All
Patient 1	7-day	316	0	1	0.0	1.2
Patient 2	7-day	296	0	0	0.0	0.0
Patient 3	7-day	239	0	2	0.0	3.1
Patient 4	7-day	667	2	0	1.1	1.1
Patient 5	10-day	369	1	0	1.0	1.0
Patient 6	7-day	737	2	1	1.0	1.5
Patient 7	7-day	982	4	2	1.5	2.2
Patient 9	7-day	946	4	0	1.5	1.5
Patient 11	7-day	452	0	0	0.0	0.0
Patient 12	7-day	331	0	0	0.0	0.0

ABR, annualized bleeding rate.
Note: Only bleeding events during prophylaxis period that occurred after dosing on or after the start date of period are counted.
[a]Duration of treatment period (days) = (end of treatment period date − start of treatment period date + 1).

 © 2024. The Author(s).
